# Drug Induced Liver Injury: Perspective of the Adverse Drug Reaction Reports to the Portuguese Pharmacovigilance System from 2010 to 2019

**DOI:** 10.3390/healthcare9121630

**Published:** 2021-11-25

**Authors:** David Ricardo da Conceição Marçal Alves Nunes, Michèle Claire Breton, Cristina Sofia de Jesus Monteiro, Jorge Luiz dos Santos

**Affiliations:** 1 Faculdade de Ciências da Saúde, Universidade da Beira Interior, 6200-506 Covilhã, Portugal; csjmonteiro@sapo.pt (C.S.d.J.M.); jlsantos@fcsaude.ubi.pt (J.L.d.S.); 2 CICS-UBI-Health Sciences Research Centre, Faculdade de Ciências da Saúde, Universidade da Beira Interior, 6200-506 Covilhã, Portugal; michele.breton20@gmail.com; 3 UFBI—Pharmacovigilance Unit of Beira Interior, Faculdade de Ciências da Saúde, Universidade da Beira Interior, 6200-506 Covilhã, Portugal

**Keywords:** drug induced liver diseases, adverse drug reaction reporting systems, pharmacovigilance system, product surveillance, post-marketing

## Abstract

Background: Drug induced liver injury (DILI) is an adverse drug reaction that causes liver damage in a predictable (dose-dependent) or an unpredictable (idiosyncratic) fashion. We performed an assessment of DILI in Portugal, by analyzing the reports, sent to the Portuguese Pharmacovigilance System (PPS). Methods: A search was performed on the PPS database, in a 10-year time frame, from 1 January 2010 to 31 December 2019. Results: There was not a prevalence of either sex in any age group. Most reports (*n* = 1120, 55.0%) belonged to patients in the age group 19–64 years old. Hepatitis (*n* = 626, 26.7%) was the most common adverse drug reaction in our study. Hepatotoxicity (*n* = 362, 15.5%) and hepatitis (*n* = 333, 14.2%) were more frequent in age group 19–64 years old. Cholestasis was more prevalent in adults independently of age. Hepatic fibrosis and encephalopathy were more common in the elderly. Most patients consumed between one and four suspected drugs (*n* = 1867, 92%). Most patients in our study evolved to “cure” (*n* = 796; 39%). Hepatotoxicity (*n* = 23; 13.8%) and hepatitis (*n* = 610; 25.9%) had a female predominancy while choluria (*n* = 8; 4.8%) and splenomegaly (*n* = 8; 4.8%) were of male predominance. Conclusions: DILI is rare but can be fatal. As such, an active search of DILI is necessary.

## 1. Introduction

Drug induced liver injury (DILI) has a low incidence in the clinical environment [1,2,3]. However, since it is frequently associated with high morbimortality levels, it is an extremely relevant subject for doctors, other health professionals, and also the pharmaceutical industry [4].

DILI can be caused by pharmaceutical drugs, herbal medicines, and food supplements [5,6]. Its incidence is hard to estimate, nevertheless, some studies point to between 14–19 cases per 100,000 population [1,2,3]. There are several difficulties in estimating DILI’s incidence since it is a diagnosis of exclusion, there are no objective diagnostic tests and, usually, adverse drug reactions (ADR) are under-reported to pharmacovigilance systems [1,2,3,6].

DILI can mimic any kind of liver disease [3,6]. The range of symptoms and signs of DILI is quite broad, ranging from asymptomatic elevation of liver enzymes to acute liver failure [2]. Furthermore, DILI is responsible for 3–5% of cases of jaundice that need hospital care and for more than 50% of cases of acute liver failure [1,2,3,7]. DILI leads to a mortality rate of around 10% [7].

Additionally, during drug development, DILI is one of the main reasons for new medicines’ non-approval in clinical trials, black boxes warnings, and recall from the marketplace [2,3,4,7,8].

DILI can be classified as direct, namely intrinsic injury, or as idiosyncratic injury [1,6].

Direct injury is dose-dependent [1,9,10], predictable [9,10], presents a short latency time [1], and its effects can be induced in animal models [1]. The most paradigmatic example of this type of injury is acetaminophen intoxication by overdose [5,6].

Idiosyncratic injuries, instead, is not dose-dependent [1,9,10,11], and thus it is unpredictable [9,10,11], presents variable latency periods, from a few days to some years [1], and their effects cannot be reproduced in animal models [1].

The aforementioned differential features of DILI types explain why most DILIs are idiosyncratic [6,9].

The number of drugs that cause hepatotoxicity has increased worldwide [5], as well as the number of publications related to the issue indexed in PubMed [12]. During drug development, a drug may not show evidence of hepatotoxicity, due to several reasons such as limited predictive value assays, lack of a validated biomarker, etc., and when the drug is already on the market (Phase IV), a DILI ensues [12]. As such, spontaneous reporting of adverse reactions is essential to detect safety issues related to drugs that escaped previous assessment, especially the idiosyncratic type due to its unpredictability [12].

The pharmacovigilance systems have an important role in detecting, registering, and evaluating ADRs [13,14,15]. Adverse reactions have high costs economically as well as socially and individually [14,16]. They require the patient to stop taking the suspected medication and increase the use of health services [14].

ADRs have a significant morbimortality, and thus a considerable impact on public health [17]. They are responsible for 5% of hospital admissions [15] and cause 197,000 deaths/year in the European Union [15].

As such, pharmacovigilance has become central in terms of detecting ADRs of both new and common-use drugs [13].

Although the effectiveness and safety of drugs are essential, some ADRs are frequently detected only in Phase IV [13].

The objective of this work was to assess DILI in Portugal in a 10-year period, from 2010 to 2019, by analyzing the reports of DILI sent to the Portuguese National Pharmacovigilance System (PPS), which is coordinated by INFARMED—National Authority of Medicines and Health Products, I.P.

## 2. Materials and Methods

### 2.1. Study Design and Ethics

This retrospective study analyzed the reports sent to PPS with at least one liver-related ADR, between 1 January 2010 to 31 December 2019. Reports were anonymous and thus Ethical committee approval was deemed unnecessary. Nonetheless, we requested it from the Ethics Commission of the Universidade da Beira Interior, and it was approved on the 28th of April 2020 with the number CE-UBI-Pj-2020-038.

### 2.2. Liver Adverse Drug Reactions

For ADRs to occur, two conditions needed to be met: (1) the drug has to cause a noxious effect; (2) the effect was not deliberated [17]. For an ADR to be classified as serious, its outcome has to be one of the following: (1) significant incapacitating disability, including birth defects; (2) hospitalization, or (3) increased hospitalization time, (4) life-threatening illness, or (5) death [18].

The definitions and terminology related to DILI used in our study came from Hoofnagle 2019 [1]; Katarey 2016 [2]; DILI: current status and future directions for drug development and the post-market setting. A consensus by a CIOMS Working Group 2020 [3]; and EASL Clinical Practice Guidelines: drug-induced liver injury 2019 [5].

We reviewed the summary of product characteristics (SmPC) of each suspected drug on the reports that contained the term “off-label use”.

### 2.3. Source and Information Contained in Reports

Each report had information corresponding to a single patient, although each report could have one or more ADRs and include one or more suspected drugs. These reports were sent to PPS either directly, by doctors, nurses, pharmacists, patients, or others health professionals, or indirectly by the marketing drug authorization holders (MAH).

Age and sex were the only demography variables available for analysis. Liver ADRs were analyzed to characterize the type, frequency, seriousness, and outcome of each DILI, including hospitalization and death.

The evolution of the patient was evaluated in reports with the following terms: cure, cure with sequels, in recovery, no recovery, death, and unknown.

The Anatomical Therapeutic Chemical (ATC) classification system allows comparisons between drug utilization studies to be made. This classification system has been recommended by WHO since 1981. It has become the gold standard to be used in fields of drug utilization, monitoring, and research [19]. As such, we assessed the number of suspected drugs and from which ATC group they belonged for each report.

The frequency of the following terms was also assessed: off-label use, overdose, and medical error.

Regulator authority assessment concerning causality of reports was also evaluated. The causality assessment belongs to the PPS and was made by experts from the PPS. We compiled the causality made by the PPS of all the reports related to DILI that were received by the PPS between 2010 and 2019. The authority classified the reports using the terms of the WHO-Uppsala Monitoring Centre for causality assessment. Terms used were as follows: certain, probable/likely, possible, unlikely, conditional/unclassified, unassessable/unclassifiable [20].

### 2.4. Report Selection

A search was performed on the PPS database, using Standardized MedDRA Queries (SMQs) related to DILI, previously selected by the authors (as seen on supplementary List S2). This search was conducted in a 10-year time frame, from 1 January 2010 to 31 December 2019. Initially, 2896 reports were obtained, of which 83 were considered invalid, 773 were duplicates and 2 were clinical trials, all these were withdrawn. After this first selection, remained 2038 reports, which were further analyzed. Data were stored in Excel files.

### 2.5. Statistical Analysis

The statistical analysis was performed using SPSS 27.0 (IBM, Portsmouth, UK). Descriptive statistical methods were used to count the data and the results were expressed as either percentage or constituent ratios. A Pearson’s Chi-square test was used to assess the association between the following variables age groups, sex, causality, death rates, and the number of suspected drugs. The Kruskal–Wallis test was used to assess for age group variables, ADRs, the evolution of each case, and the number of suspected drugs. The studied variables were reported to the PPS. *p*-values < 0.05 were considered significant for both tests.

## 3. Results

According to the last census data in Portugal, which was concluded in 2011, the total population was 10,562,178 inhabitants. The ratio between males to females was 0.9149. There were 1,572,329 individuals in the age group 0–14 years old, 6,979,785 individuals in the age group 15–64 years old, and 2,010,064 individuals older than 64 years [21]. More recent demographic data related to the Portuguese population has not been concluded due to the current COVID-19 pandemic [21].

**Figure 1:**
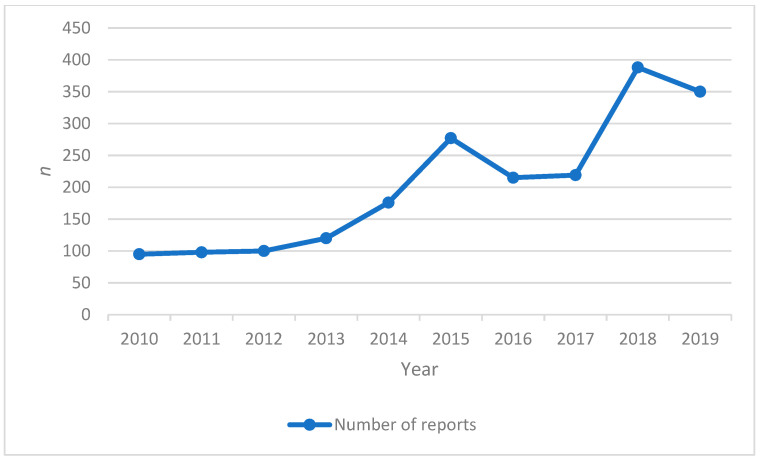
Temporal evolution of the number of reports related to DILI.

In the period between 1 January 2010 to 31 December 2019, the PPS received 2038 reports related to liver ADRs. As shown in Figure 1, there was an increasing trend over time in the number of reports received by the PPS. The 3 years with the highest number of reports (2015, 2018–2019) correspond to almost half (49.8%) of the total number of reports. 

Table 1 presents the demographic distribution of liver ADRs.

Table 1 shows reports from male (*n* = 980, 48.1%) and female (*n* = 968, 47.5%) patients. In *n* = 90 (4.4%) reports, patient sex was uninformed. Regarding age distribution, the youngest individual was 1 year old and the oldest was aged 96 years at the occurrence of ADR. The age group 19–64 years included most ADRs, comprising *n* = 1120 (55%) cases, involving males (*n* = 542, 26.6%) and females (*n* = 563, 27.6%). The age group 1–3 years presented with the least number of reports, in total (*n* = 21, 1.0%), in males (*n* = 12, 0.6%) and in females (*n* = 8, 0.4%).

Table 2 presents the distribution of liver ADRs according to age groups.

Table 2 shows that the most frequent ADR was hepatitis (*n* = 626, 26.7%) and the least was acholic stools (*n* = 27, 1.1%). Acute ADRs, such as hepatotoxicity (*n* = 362, 15.5%) and hepatitis (*n* = 333, 14.2%) were more prevalent in age group 19–64 years. Cases of cholestasis were more prevalent in adults, irrespective of age group, 19–64 (*n* = 101, 4.3%) and ≥64 years (*n* = 80, 3.4%). Hepatic fibrosis (*n* = 97, 4.1%) had the highest prevalence in patients aged 19–64 years, and encephalopathy (*n* = 12, 0.5%) had the highest prevalence in patients over 64 years old. 

The three most frequently altered laboratory tests were: aminotransferases (*n* = 494, 24.2%), more specifically ALT (*n* = 244, 12%) and bilirubin (*n* = 293, 14.4%). Prothrombin time (*n* = 41, 2%) was the least frequently altered. Aminotransferases increases were more pronounced in adults, age group 19–64 (*n* = 264, 13.0%) and ≥64 years (*n* = 101, 5.0%). Laboratory markers of cholestasis, such as GGT, had higher frequencies in age group 19–64 (*n* = 117, 5.7%) and ≥64 years (*n* = 67, 3.3%), and alkaline phosphatase was also more prevalent in age group 19–64 (*n* = 65, 3.2%) and ≥64 years (*n* = 50, 2.5%). In age groups corresponding to children and adolescents, there was a low frequency of laboratory results mentioned in the reports. 

A high prevalence of drugs used as off-label was observed in the age group 19–64 years (*n* = 49, 2.4%). Regarding the total of 78 cases of off-label use, in which there had been an ADR, 6.4% occurred in the pediatric population and had no indication for their use, 8.9% had been used in accordance with indication, but with higher doses than approved on the SmPC. The remaining 84.7% occurred in adults and resulted from the use of drugs in conditions without indication on the SmPC. 

Supplementary Table S1 shows that the highest prevalence of off label used medication was for onychomycoses (*n* = 5, 6.41%), followed by abdominal wall hematoma (*n* = 4, 5.13%). Others with equally high prevalence (*n* = 3, 3.85%) were: thalamic pain; chronic hepatitis C; thyrotoxicosis, and bradycardia as well as chronic hepatitis C and HIV co-infection.

Supplementary Table S2 displays the distribution of liver ADRs causality to the number of suspected drugs. The causality assessment belongs to the PPS and was made by experts from the PPS. Of the initial 2038 reports, only 1828 were serious to merit an assessment. The highest number of reports had the “unassigned” category attributed (*n* = 1303, 71.3%) and the category “definitive” (*n* = 24, 1.3%) had a low prevalence. Other categories had values ranging from (*n* = 273, 14.9%) for “likely” to (*n* = 3, 0.2%) for “not classifiable”.

Table 3 presents the liver ADRs distribution in relation to the number of suspected drugs. 

Table 3 shows that acute cases of hepatopathy, such as hepatitis, hepatotoxicity, and jaundice, were observed in patients who consumed one to four drugs suspected of causing ADRs. On the other hand, hepatic fibrosis was more common in patients taking between five to nine suspected drugs. Pruritus, autoimmune hepatitis, and choluria were more common in those patients who consumed one to four suspected drugs.

Table 4 shows the distribution of clinical outcomes to the number of drugs suspected to have caused liver ADRs.

Table 4 Most reports included between one and four suspected drugs (*n* = 1867, 92%). Concerning patients’ clinical evolution, most patients had a favorable outcome, as “cured” (*n* = 796, 39%) and “in recovery” (*n* = 295, 15%). Death was reported in *n* = 126 (6.2%) patients, “no recovery” in *n* = 105 (5.2%), whereas *n* = 25 (1.2%) were “cured with sequels”. The highest number of patients that were in categories “cured” (*n* = 746, 36.6%) and “in recovery” (*n* = 285, 14.0%), had taken between one and four medications.

Table 5 depicts the distribution of liver ADRs to the number of suspected drugs concerning the patients who died.

As seen on Table 5, among patients who died (*n* = 126), most were male (*n* = 70, 55.6%). Concerning the number of ADRs of patients who died (*n* = 167, 100%) in total, the majority occurred in males (*n* = 106, 63.5%). The most frequent liver ADRs of the patients who died were hepatotoxicity (*n* = 46, 27.5%), followed by hepatitis (*n* = 37, 22.2%) and encephalopathy (*n* = 25, 15%). Regarding the sex distribution, both hepatotoxicity (*n* = 23, 13.8%; *p* = 0.006) and hepatitis (*n* = 21, 12.6%; *p* = 0.001) were more prevalent in females, whereas male predominance occurred in both choluria (*n* = 8, 4.8%; *p* = 0.031) and splenomegaly (*n* = 8, 4.8%; *p* = 0.031). 

Supplementary Table S3 shows the distribution and characteristics of cases with positive viral markers. It depicts a low frequency of viral positivity (*n* = 31, 1.5%) in our study. The viruses were by decreasing order of frequency: hepatitis C virus (*n* = 10, 0.49%), cytomegalovirus (*n* = 6, 0.29%), hepatitis B virus (*n* = 5, 0.25%), both herpes zoster virus (*n* = 3, 0.15%) and HIV (*n* = 3, 0.15%) had the same frequency, Epstein–Barr virus (*n* = 2, 0.1%) and finally both hepatitis E virus (*n* = 1, 0.05%) and herpes simplex (*n* = 1, 0.05%) with the same frequency.

Supplementary Table S4 presents the distribution of the reported cases of ADRs according to the ATCC classification. It shows that there were 3293 (100%) suspected drugs. The most frequent ATC groups were, by descending order: J05—antivirals for systemic use (*n* = 713, 21.65%), L01—antineoplastic agents (*n* = 430, 13.06%), L04—immunosuppressive agents (*n* = 344, 10.45%), J01—antibacterial agents for systemic use (*n* = 282, 8.56%) and N05—psycholeptic agents (*n* = 186, 5.65%).

## 4. Discussion

This study investigated the picture of DILI under the perspective of the ADRs reports informed to the PPS in the last decade.

Table 1 does not demonstrate a prevalence of either sex in any age group for DILI. This finding agrees with the EASL Clinical Practice Guidelines [5] in that “sex does not appear to be a general risk factor for DILI”. Other studies [22,23] also did not find a relation between sex and increased incidence of DILI. 

Age, on the other hand, is generally accepted as a risk factor for DILI [7]. For instance, in causality assessment methods used for DILI, namely the RUCAM, or the CIOMS scale, age constitutes a risk factor, as people over 55 years of age are attributed 1 extra point [3,11,24]. In this study (Table 1), age was not a risk factor for DILI, which is in line with data from large DILI registries in Spain and the USA [25,26]. Since there is increasing evidence suggesting that the elderly are more susceptible to certain drugs [22,25], it is inferable that age may function as a contributing factor. 

Acute cases of liver ADRs, such as hepatotoxicity and hepatitis were found to be more frequent in adults with less than 65 years old (Table 2). Considering the clinical descriptions used in the evaluated reports, the term hepatotoxicity seemed to refer to acute hepatitis. Hepatitis can be caused by virus [22,23,26,27], autoimmune diseases [23,26], drugs [23] and some genetic metabolic diseases [28]. In this study, there was a low prevalence of hepatotropic virus (Supplementary Table S3), which may indicate a low frequency of viral hepatitis in the evaluated sample. In this study, neither autoimmune nor genetic metabolic diseases were evaluated.

Cholestasis and its laboratory markers, GGT and ALP, were more prevalent in patients above 18 years of age (Table 2). This finding is in agreement with other studies, in which it was also observed that cholestatic pattern of injury had a higher occurrence in older patients [22,23,26,27,29]. Certain drugs have a higher tendency to cause a cholestatic pattern of liver injury than others [22,30]. For instance, drugs that are excreted via bile are more likely to induce cholestatic liver injury in susceptible patients [29]. Drugs known to be associated with cholestatic liver damage include several agents with different properties, such as antibiotics, anti-inflammatory drugs, psychotropics drugs, anticonvulsants, statins, immunosuppressants, and hypoglycemic drugs [23,30]. Cholestatic liver injury can be due to mixed hepatocellular cholestatic damage or to an alteration of bile flow in bile canaliculi, resulting in pure canalicular cholestasis and even in obstructive cholangiopathy [29,31,32]. Canalicular cholestasis can result from the use of anabolic steroids and estrogens [29]. Other causes of cholestasis such as biliary mechanical obstruction, primary biliary cholangitis, primary sclerosing cholangitis, viral hepatitis, alcoholic and non-alcoholic liver disease, gestational cholestasis, genetic-metabolic disorders, associated with different age groups should be excluded [29,33]. There was a low prevalence of viral hepatitis (Supplementary Table S3), meaning a low likelihood for this type of etiology. Genetic variations of liver transport proteins between patients could also explain why some individuals are more susceptible than others to cholestatic injury [29]. Cholestatic idiosyncratic DILI reactions are unpredictable and result from immune-mediated biliary disruption [1,8]. Drug-protein adducts are formed and are presented as a new type of antigen, which leads to the immune reaction [8]. Patients that harbor alleles HLA-DBR1*15 and HLA-DQB1*06 seem to have a higher propensity to develop cholestatic DILI [27], and certain human leukocyte antigens (HLA) play a significant role in this type of injury [34].

Hepatic fibrosis was highest in the age group 19–64 years (Table 2). Hepatic fibrosis occurs when there is an alteration in the process of the wound healing response to chronic liver damage that favors the increased deposition of extracellular matrix proteins, including fibrillar collagens [35]. Alcohol, hepatotropic virus, and non-alcoholic steatohepatitis are among the most common causes of hepatic fibrosis [35]. Supplementary Table S3 shows a low frequency of viral markers, as such these viruses had a low impact on our study. However, it is possible that the hepatic fibrosis described in some patients of this study resulted from non-diagnosed primary chronic liver diseases.

In our study, encephalopathy had the highest frequency in patients over the age of 64 years (Table 2). Encephalopathy can be caused by several factors, such as metabolic alterations, brain atrophy, brain edema, and liver failure [36]. Given that the reports under analysis were selected using keywords related to liver disease, it is likely that encephalopathy was caused by hepatic failure [36], but the influence of additional causes was not ascertained. Fulminant liver failure leads to death or the need for liver transplantation [22]. It occurs more frequently in females that harbor a hepatocellular pattern of injury [22,27]. Most cases of fulminant liver failure are attributable to hepatotoxicity caused by acetaminophen, whereas the second leading cause is idiosyncratic DILI [22]. The ratio of acute liver failure due to acetaminophen has risen over the years and most cases are due to unintentional overdoses [22], although, suicide attempts must be excluded [22]. We did not ascertain if DILI cases were idiosyncratic or intrinsic because the reports neither mentioned the terms nor had enzyme values to calculate the R-value. As such, we could not identify and exclude any reports that might have intrinsic DILI. This might have an effect on our study conclusions. We did not find any mention of possible suicide attempts on the reports analyzed. Drugs that have more than 50% of their metabolism executed by the liver have a higher likelihood of fulminant liver failure [27]. There is also a significant relationship between high oral drug doses and the higher likelihood of liver failure [27]. In this study, neither the site of drug metabolization nor the route of administration of the medications described in the ADRs was evaluated.

Concerning the age groups of children and adolescents (Table 2), there was a low frequency of liver ADRs, and a low frequency of laboratory results mentioned in the reports. DILI is a rare occurrence in the pediatric population [37] and children do not seem to be more at risk of DILI than the rest of the population [22]. Certain drugs, especially drugs that act on the central nervous system, such as antiepileptics and psychotropics, and antimicrobials, are more frequently associated with cases of DILI in children [22,27,37]. Children are also more affected by drugs that cause a hepatocellular pattern of injury [22,37]. However, as mentioned before, we were not able to assess whether DILI was idiosyncratic or intrinsic. Nonetheless, most cases of DILI in children are scored as either mild or moderate [37]. Another possibility for the findings observed in Table 2 was under recognition, and thus underreporting of DILI in those age groups. However, the effects of childhood particularities regarding drug pharmacokinetics [37] could not be excluded in the present study.

Table 2 shows that drugs used as “off label” had a high prevalence among adult patients younger than 65 years old. “Off label” use of drugs occurs commonly [38]. “Off label” prescription results from the use of different drugs of the same class and with similar effects, therapeutic attempts when additional therapies have failed, or in populations for which a specific drug use is not yet approved [38]. In the present study, the justification of the “off label” use of medications was not analyzed.

Concerning causality assessment (Supplementary Table S2), which belongs to the PPS and was made by experts from the PPS. Supplementary Table S2 describes the causality to all the reports related to DILI received between 2010 to 2019 by the PPS. The data shown does not represent a sample of the data, but all the data available. According to PPS, it is only mandatory for experts to assess serious ADRs. Moreover, the pharmaceutical industry only has to make a causality assessment if death occurs if there is a risk of life, or congenital anomaly. As most of the reports analyzed in this study came from the pharmaceutical industry, it explains why most analyzed reports did not have causality attributed and belonged to the category “unassigned”. It is worth highlighting that the conclusions from our study are in agreement with conclusions from the EASL Clinical Practice Guidelines [5] and from data from large DILI registries in Spain and the USA [25,26]. This means that the use of information collected by the pharmacovigilance system can effectively be used to find new possible relations between different variables.

Acute cases, including hepatitis, hepatotoxicity, jaundice, and choluria were more frequent in patients that used between one to four suspected drugs (Table 3). This may be related to the fact that these patients might have taken drugs that caused intrinsic DILI. As this type of injury has a short latency time [8].

Hepatic fibrosis was more common in patients taking more than four suspected drugs (Table 3). Certain drugs can cause cholestasis [22,30], and cholestasis can evolve into hepatic fibrosis [35]. Which could explain our study results.

Pruritus was more common in patients that had taken less than five suspected drugs (Table 3). There are several causes of pruritus, such as hepatic cholestasis, renal failure, dermatological causes, drugs, iron deficiency anemia, thyrotoxicosis, oncologic diseases, among others [39,40]. In the present study, the cause of pruritus, if associated with cholestasis or not, could not be ascertained.

Autoimmune hepatitis (AIH) was described in 64 (2.7%) patients in Table 3. AIH is a cause of chronic liver disease with different triggers including prescription drugs, viral infections, associated systemic autoimmune disorders, and liver transplant [41]. Given the low prevalence of viral markers positivity, or of liver transplants, in this study, other factors should explain the occurrence of AIH observed. In this study, we could not identify if AIH was the primary disease or was caused by the suspected drugs under report. Contrary to hepatic fibrosis, autoimmune hepatitis was more common in people that had taken less than five suspected drugs, suggesting that if AIH was triggered by drugs it may have not been triggered by drug interactions.

Concerning the number of suspected drugs used and the cases evolution (Table 4), few reports included more than five suspected drugs in use. Most patients presenting DILI received between one to four suspected drugs. In categories “cure” and “in recovery”, most patients had taken less than five suspected drugs. The higher number of drugs can result in drug interaction leading to DILI aggravation and lower frequencies of cure or recovery [27,42,43].

Table 5 showed that in patients who died, there were more females than males with hepatitis and hepatotoxicity. Choluria and splenomegaly, features of cholestasis and portal hypertension had more male representation than female. This finding agrees with other studies showing that females have a higher tendency for hepatocellular damage, while cholestatic disorders occur more often in males than females [22,27].

In this study, we used the ATC classification system (Supplementary Table S4) to ascribe the drugs related to liver ADRs. We obtained ATC codes in every first level, with the notable exception of sensory organs. The five most frequent drugs belonged to the following groups by descending order: antivirals for systemic use (most were used in HIV/AIDS patients), antineoplastic agents, immunosuppressive agents, antibacterial agents for systemic use, and psycholeptic agents, both included in the group of drugs that cause idiosyncratic DILI [21]. In the EASL Clinical Practice Guidelines [5], it is referred that the following drugs are associated with idiosyncratic DILI: antimicrobials, central nervous system, cardiovascular, immunomodulatory, antineoplastic, rheumatologic. If we consider that antivirals for systemic use are in the same category as antimicrobials, our study results are in agreement with the EASL Clinical Practice Guidelines [5]. However, we had few cases of cardiovascular drugs related to DILI. This might either be explained by a difference in population genetics or local variations of prescription patterns [5,27].

Regarding our study’s limitations, most of them are attributable to the low quality of information and lack of essential clinical-laboratory data obtained from the ordinary reports of ADRs to the PPS. Reports were not filled completely, sometimes relevant patient information was missing, such as age and sex. Laboratory tests were only mentioned in reports as either altered, increased, or decreased. As such, it was impossible to ascertain the degree of change from normality. For the same reason, it was also not possible to qualify the type of DILI as either hepatocellular, cholestatic or mixed. Another limitation was the high number of serious reports that had not been assessed for causality. Possible influences in this study results are other drugs, not suspected of causing ADRs, over-the-counter medication, alcohol consumption, herbal products, and food supplements. These variables were not mentioned in the reports and their possible impact on our study results should be taken into account.

## 5. Conclusions

In conclusion, pharmacovigilance systems are extremely important to assess the existence, frequency, and seriousness of putative ADRs that are only known when a drug is administered to a large population.

Experiences made by regional, as well as, national pharmacovigilance systems are valuable, as they allow analyses of observed DILI frequency. They also put in evidence methodological challenges faced by researchers when making assessments and trying to understand ADRs’ causal relationships between drugs and clinical outcomes, in this case, liver related. Only after taking into account these difficulties, will it be possible to implement adjustments with the intent to improve ADRs reports’ quality as well as of the system as a whole. These improvements will allow the collection of more precise epidemiological data, which in turn, will allow a better implementation of evidenced based preventive public policies, as these are closer to reality.

DILI is a rare occurrence, although it can be serious and sometimes even fatal. As such, continuous monitoring of liver adverse drug reactions is necessary. In our study, hepatitis was the most common liver ADR and most patients had a full recovery.

In Portugal, DILI pharmacovigilance studies are scarce. As such, it was our primary goal to increase the public health base of knowledge concerning DILI.

To decrease the incidence of DILI, we would advise following these recommendations.

In wards, at the hospital level, drugs known to cause DILI in a significant way as well as liver enzymes of patients taking drugs should be monitored to prevent DILI from occurring, or at least that DILI is not serious.

At the community pharmacy level, we propose decreasing the availability of drugs sold as over-the-counter medications that are known to cause DILI in a significant way. This could be achieved by either decreasing doses available or by reducing the concentration per tablet. Higher doses would still be available, but only with medical prescriptions.

This study highlights the need to provide orientation for health professionals about the importance of writing reports as formally and detailly as possible. Our study concerns, not just hepatopathies caused by drugs on a national level as is the case in Portugal, but it discusses the challenges of ADRs reports, and the conclusion drawn here can be extrapolated to international levels.

## Figures and Tables

**Table 1:** Distribution of the reports of hepatic adverse drug reactions, according to age groups and sex.

Age Group		Sex			Χ^2^	*p*
	Female *n* = 968 *n* (%)	Male *n* = 980 *n* (%)	NI ^2^ *n* = 90 *n* (%)	Total *n* = 2038 *n* (%)		
1–3 years	8 (0.4)	12 (0.6)	1 (0.0)	21 (1.0)	0.75	0.383
4–12 years	25 (1.2)	24 (1.2)	2 (0.1)	51 (2.5)	0.02	0.850
13–18 years	18 (0.9)	13 (0.6)	5 (0.2)	36 (1.8)	0.83	0.347
19–64 years	563 (27.6)	542 (26.6)	15 (0.7)	1120 (55.0)	0.37	0.203
>64 years	202 (9.9)	203 (10.0)	11 (0.5)	416 (20.4)	0.01	0.933
NI ^1^	152 (7.4)	186 (9.7)	56 (2.7)	394 (19.3)		

Abbreviations: NI—not informed. ^1;2^—data not included in the statistical analysis. White spaces mean no data. Pearson’s Chi-squared test and Kruskal–Wallis test were used.

**Table 2:** More frequently reported hepatic adverse drug reactions, according to age groups.

Adverse Reaction	Age Group *n* (%)								
	1–3 Years (*n* = 21)	4–12 Years (*n* = 51)	13–18 Years (*n* = 36)	19–64 Years (*n* = 1120)	>64 Years (*n* = 416)	NI ^1^ (*n* = 394)	Total (*n* = 2038)	Χ^2^	*p*
Hepatitis	8 (0.3)	16 (0.7)	14 (0.6)	333 (14.2)	164 (7.0)	91 (3.9)	626 (26.7)	13.98	0.007
Hepatotoxicity	5 (0.2)	10 (0.4)	5 (0.2)	362 (15.5)	114 (4.9)	104 (4.4)	600 (25.6)	11.60	0.020
Jaundice	6 (0.3)	7 (0.3)	4 (0.2)	142 (6.1)	67 (2.9)	37 (1.6)	263 (11.2)	7.13	0.129
Cholestasis	3 (0.1)	6 (0.3)	8 (0.3)	101 (4.3)	80 (3.4)	32 (1.4)	230 (9.8)	34.07	<0.001
Rash	1 (0.0)	4 (0.2)	2 (0.1)	62 (2.6)	21 (0.9)	13 (0.6)	103 (4.4)	0.72	0.947
Hepatic fibrosis				97 (4.1)	1 (0.0)	1 (0.0)	99 (4.2)	45.69	<0.001
Ascites			2 (0.1)	45 (1.9)	24 (1.0)	26 (1.1)	97 (4.1)	5.74	0.218
Pruritus		6 (0.3)	2 (0.1)	60 (2.6)	21 (0.9)	8 (0.3)	97 (4.1)	5.33	0.254
Autoimmune hepatitis		2 (0.1)		43 (1.8)	11 (0.5)	8 (0.3)	64 (2.7)	3.42	0.489
Choluria	1 (0.0)	2 (0.1)	2 (0.1)	26 (1.1)	15 (0.6)	2 (0.1)	48 (2.1)	3.47	0.481
Encephalopathy		1 (0.0)	2 (0.1)	2 (0.1)	12 (0.5)	30 (1.3)	47 (2.0)	29.54	<0.001
Cirrhosis				28 (1.2)	5 (0.2)	8 (0.3)	41 (1.8)	4.96	0.290
Acholic stool		1 (0.0)		13 (0.6)	13 (0.6)		27 (1.1)	8.25	0.082
**Laboratory tests**									
Aminotransferase	2 (0.1)	18 (0.9)	8 (0.3)	264 (13.0)	101 (5.0)	101 (5.0)	494 (24.2)	6.18	0.186
Bilirubin	5 (0.2)	5 (0.2)	2 (0.1)	153 (7.5)	73 (3.6)	55 (2.7)	293 (14.4)	8.46	0.076
ALT	4 (0.2)	4 (0.2)	5 (0.2)	130 (6.4)	70 (3.4)	31 (1.5)	244 (12.0)	9.23	0.055
AST	5 (0.2)	4 (0.2)	3 (0.1)	112 (5.5)	71 (3.5)	26 (1.3)	221 (10.8)	18.57	<0.001
GGT	4 (0.2)	3 (0.1)	1 (0.0)	117 (5.7)	67 (3.3)	35 (1.7)	227 (11.1)	15.08	0.004
Alkaline phosphatase	2 (0.2)	3 (0.1)		65 (3.2)	50 (2.5)	16 (0.8)	136 (6.7)	20.53	<0.001
Lactate dehydrogenase	1 (0.0)			49 (2.4)	21 (1.0)	6 (0.3)	77 (3.8)	4.48	0.344
Prothrombin time				34 (1.7)	6 (0.3)	1 (0.3)	41(2.0)	6.12	0.189
**Procedural complications**									
Off label use	2 (0.2)	3 (0.1)	4 (0.2)	49 (2.4)	2 (0.2)	18 (0.9)	78 (3.8)	22.03	<0.001
Drug exposure Pregnancy				13 (0.6)		10 (0.4)	23 (1.1)	6.13	0.189
Overdose		1 (0.0)	1 (0.0)	12 (0.6)		2 (0.2)	16 (0.8)	6.71	0.151
Medical error				4 (0.2)	2 (0.2)	2 (0.2)	8 (0.4)	0.55	0.968

Abbreviations: ALT—alanine aminotransferase; AST—aspartate aminotransferase; GGT—gamma-glutamyl transferase; NI—not informed. ^1^—data not included in the statistical analysis. White spaces mean no data. Pearson’s Chi-squared test was used.

**Table 3:** Frequency of different hepatic adverse drug reactions reported according to the number of suspected drugs.

Number of Suspected Drugs	1–4	5–9	≤10	Total	Χ^2^	*p*
Adverse Reactions *n* (%)						
Hepatitis	610 (25.9)	12 (0.5)	4 (0.2)	626 (26.6)	68.48	<0.001
Hepatotoxicity	489 (20.8)	74 (3.1)	37 (1.6)	600 (25.5)	41.59	<0.001
Jaundice	260 (11.0)	3 (0.1)	0 (0.0)	263 (11.2)	31.87	<0.001
Cholestasis	204 (8.7)	22 (0.9)	4 (0.2)	230 (9.8)	1.94	0.378
Rash	97 (4.1)	6 (0.3)	0 (0.0)	103 (4.4)	4.70	0.095
Hepatic fibrosis	21 (0.9)	55 (2.3)	23 (1.0)	99 (4.2)	454.55	<0.001
Pruritus	93 (4.0)	0 (0.0)	4 (0.2)	97 (4.1)	9.55	0.008
Ascites	85 (3.6)	12 (0.5)	0 (0.0)	97 (4.1)	4.97	0.083
Encephalopathy	75 (3.2)	10 (0.4)	0 (0.0)	85 (3.6)	3.87	0.143
Autoimmune hepatitis	64 (2.7)	0 (0.0)	0 (0.0)	64 (2.7)	8.67	0.001
Choluria	48 (2.0)	0 (0.0)	0 (0.0)	48 (2.0)	6.45	0.039
Cirrhosis	33 (1.4)	6 (0.3)	2 (0.1)	41 (1.7)	2.53	0.281
Total	2079 (88.4)	200 (8.5)	74 (3.1)	2353 (100.0)		

Pearson’s Chi-square test was used.

**Table 4:** Clinical evolution of patients associated with the hepatic adverse reaction, according to the number of suspected drugs.

Number of Suspected Drugs	1–4 (*n* = 1867)	5–9 (*n* = 131)	≥10 (*n* = 40)	Χ^2^	*p*
Age (Mean ± SD)	52 ± 20	38 ± 16	35 ± 18		
Case Evolution *n* (%)					
Cure	746 (36.6)	36 (1.8)	14 (0.7)	8.28	0.001
In recovery	285 (14.0)	10 (0.5)	0 (0.0)	12.66	0.001
Cure with sequels	25 (1.2)	0 (0.0)	0 (0.0)	2.31	0.313
No recovery	102 (5.0)	2 (0.1)	1 (0.0)	4.46	0.107
Death	115 (5.6)	11 (0.5)	0 (0.0)	3.74	0.153
Unknown ^1^	594 (29.1)	72 (3.5)	25 (1.2)		

^1^—Data not included in the statistical analysis.

**Table 5:** Sex distribution related to the hepatic adverse reactions in the patients who died.

Adverse Reaction	Total *n* (%)	Female *n* (%)	Male *n* (%)	NI ^1^ *n* (%)	Χ^2^	*p*
Hepatotoxicity	46 (27.5)	23 (13.8)	21 (12.6)	2 (1.2)	7.51	0.006
Hepatitis	37 (22.2)	21 (12.6)	15 (9)	1 (0.6)	10.64	0.001
Encephalopathy	25 (15)	8 (4.8)	17 (10.2)	0 (0)	0.14	0.702
Jaundice	17 (10.2)	4 (2.4)	13 (7.8)	0 (0)	1.16	0.280
Ascites	14 (8.4)	2 (1.2)	12 (7.2)	0 (0)	2.97	0.084
Choluria	8 (4.8)	0 (0)	8 (4.8)	0 (0)	4.60	0.031
Splenomegaly	8 (4.8)	0 (0)	8 (4.8)	0 (0)	4.60	0.031
Cholestasis	4 (2.4)	0 (0)	4 (2.4)	0 (0)	2.24	0.134
Cirrhosis	4 (2.4)	0 (0)	4 (2.4)	0 (0)	2.24	0.134
Hepatomegaly	4 (2.4)	0 (0)	4 (2.4)	0 (0)	2.24	0.134
**Number adverse reactions**	167 (100.0)	58 (24.7)	106 (63.5)	3 (1.8)		
**Number of cases**	126	51 (40.5)	70 (55.6)	5 (4.)		
**Age (mean ± SD)**	57 ± 20	57 ± 20	57 ± 21	56 ± 00		

Abbreviations: NI—not informed. ^1^—Data not included in the statistical analysis.

## Data Availability

Restrictions apply to the availability of these data. Data were obtained from the National Authority of Medicines and Health Products, I.P. (INFARMED), Lisbon, Portugal and are available with the permission of the National Authority of Medicines and Health Products, I.P. (INFARMED), Lisbon, Portugal.

## Supplementary Materials

The following are available online at https://www.mdpi.com/article/10.3390/healthcare9121630/s1. Table S1—Drugs used off label, Table S2—Causality rates in cases of serious hepatic adverse drug reactions in relation to the number of suspected drugs, Table S3—Cases with positive viral markers included in the sample of patients with reported adverse drug reactions, and Table S4—Distribution of reported cases of adverse drug reactions according to the Anatomical Therapeutic Chemical Code (ATCC). Supplementary List S1—Acronyms, and Supplementary List S2—List of Standardized MedDRA Queries (SMQs) related to drug induced liver injury.
