# Supplementary material for: Drug Induced Liver Injury: Perspective of the Adverse Drug Reaction Reports to the Portuguese Pharmacovigilance System from 2010 to 2019
Source: Healthcare (Basel). 2021 Nov 25;9(12):1630. doi: 10.3390/healthcare9121630 (PMC8702164; doi:10.3390/healthcare9121630)
Supplement: Supplementary file 1 [file healthcare-09-01630-s001.zip › healthcare-1463327-supplementary/Supplementary List S1.pdf]

Supplementary List S1 - Acronyms

AIDS: Acquired Immunodeficiency Syndrome

AIH: Autoimmune Hepatitis

ALP: Alkaline Phosphatase

ALT: Alanine Aminotransferase

AST: Aspartate Aminotransferase

ATC: Anatomical Therapeutic Chemical Classification

CIOMS: Council for International Organizations of Medical Sciences

CMG: Cytomegalovirus

DILI: Drug Induced Liver Injury

EASL: European Association for the Study of the Liver

EBV: Epstein-Barr Virus

FC: Fibrosis Cirrhosis

FH: Fulminant Hepatitis

GGT: Gamma-Glutamyl Transferase

HBV: Hepatitis B Virus

HCV: Hepatitis C Virus

HEV: Hepatitis E Virus

HIV: Human Immunodeficiency Virus

HLA: Human Leucocyte Antigens

HM: Hepatomegaly

IH: Indication for Hospitalization

INFARMED: National Authority of Medicines and Health Products, I.P.

n: Number of Cases

ND: Not Described

NI: Not Informed

NR: Not Relevant

PPS: Portuguese Pharmacovigilance System

RAM: Reação Adversa a Medicamento

RUCAM: Roussel Uclaf Causality Assessment Method

SmPC: Summary of Product Characteristics

SMQs: Standardised MedDRA Queries

SPSS: Statistical Package for the Social Sciences

USA: United States of America

WHO: World Health Organization
