# Supplementary material for: Drug Induced Liver Injury: Perspective of the Adverse Drug Reaction Reports to the Portuguese Pharmacovigilance System from 2010 to 2019
Source: Healthcare (Basel). 2021 Nov 25;9(12):1630. doi: 10.3390/healthcare9121630 (PMC8702164; doi:10.3390/healthcare9121630)
Supplement: Supplementary file 1 [file healthcare-09-01630-s001.zip › healthcare-1463327-supplementary/Supplementary List S2.pdf]

**Supplementary List S2**

List of Standardized MedDRA Queries (SMQs) related to drug induced liver injury

Steatohepatitis

Non-alcoholic hepatic steatosis

Hepatitis

Acute hepatitis

Allergic hepatitis

Autoimmune hepatitis

Cholestatic hepatitis

Chronic hepatitis

Chronic active hepatitis

Chronic persistent hepatitis

Fulminant hepatitis

Immune-mediated hepatitis

Toxic hepatitis

Inflammation of the portal tract

Ascites

Acute yellow liver atrophy

Liver atrophy

Biliary cirrhosis

Hepatic cirrhosis

Primary biliary cholangitis

Hepatic coma

Acquired hepatocerebral degeneration

Liver disease

Non-alcoholic Fatty Liver Disease

Hepatobiliary disease

Hepatic encephalopathy

Minimal hepatic encephalopathy

Steatohepatitis

Non-alcoholic hepatic steatosis

Biliary fibrosis

Fibrosis and hepatic steatosis

Liver fibrosis

Portal fibrosis

Damaged liver

Fulminant hepatitis

Hepatotoxicity

Non-cirrhotic portal hypertension

Eosinophilic hepatic infiltration

Liver failure

Acute liver failure

Chronic liver failure

Subacute liver failure

Hepatorenal failure

Cholestatic liver damage

Liver damage

Drug-induced liver injury

Mixed liver injury

Liver necrosis

Reye's Syndrome

Hepatopulmonary syndrome

Hepatorenal syndrome

Acute outbreak of chronic liver failure

Liver transplant

Hepatobiliary cancer

Mixed hepatocellular cholangiocarcinoma

Cholangiosarcoma

Alteration of bilirubin excretion

Cholestasis

Parenteral nutrition-associated liver disease

Cholestatic hepatitis

Hyperbilirubinemia

Jaundice

Cholestatic jaundice

Hepatocellular jaundice

Eye jaundice

Increased jaundice index

Cholestatic liver damage

Mixed liver injury

Cholestatic itching

Bile secretion deficiency

Yellow skin

Increased Total Bile Acids

Alanine aminotransferase abnormal

Alanine aminotransferase increased

Increased ammonia

Ascites

Abnormal aspartate aminotransferase

Increased Aspartate Aminotransferase

Increased mitochondrial aspartate aminotransferase

Abnormal conjugated bilirubin

Increased conjugated bilirubin

Increased direct blood bilirubin

Increased urine bilirubin

Abnormal bilirubin in blood

Increased bilirubinemia

Abnormal liver biopsy

Abnormal bile flow

Decreased bile flow

Abnormal liver ultrasound

Abnormal liver enzyme

Increased liver enzyme

Palpable liver

Abnormal liver function

Gamma glutamyltransferase abnormal

Increased gammaglutamyltransferase

Hepatosplenomegaly

Hepatomegaly

Hypermonemia

Hyperbilirubinemia

Hypercholia

Hypertransaminasemia

Hepatic hypertrophy

Magnetic resonance imaging of abnormal liver

Presence of bilirubin in urine

Abnormal liver function test

Increased liver function test

Abnormal AST/ALT ratio

Abnormal liver scan

Abnormal hepatobiliary scan

Abnormal Transaminases

Increased Transaminases

5'-nucleotidase increased

Bile secretion deficiency

Abnormal blood alkaline phosphatase

Increased blood alkaline phosphatase

Increased urinary urobilinogen

Decreased urinary urobilinogen
