# Supplementary material for: Drug Induced Liver Injury: Perspective of the Adverse Drug Reaction Reports to the Portuguese Pharmacovigilance System from 2010 to 2019
Source: Healthcare (Basel). 2021 Nov 25;9(12):1630. doi: 10.3390/healthcare9121630 (PMC8702164; doi:10.3390/healthcare9121630)
Supplement: Supplementary file 1 [file healthcare-09-01630-s001.zip › healthcare-1463327-supplementary/Supplementary Tables.pdf]

Supplementary Table S1

Drug	n	off label use
Amoxicillin plus clavulanic acid.	4	Abdominal wall hematoma.
Eculizumab	1	Glomerulonephritis crescentic.
Idealisib	1	Chronic lymphocytic leukaemia as first-line therapy
imatinib	1	Severe myelofibrosis.
immunoglobulin G	1	Atopic dermatitis.
Amiodarone and esmolol	2	Bradycardia
Azithromycin	1	Mybobacterium lentiflavum.
Baclofen	3	Thalamic pain.
Budesonide (gastro-resistant capsule) and esomeprazole.	1	Respiratory tract infection.
Buprenorphine.	3	HIV disease, HCV coinfection and chronic hepatitis C.
Bupropion	1	Depressive symptoms and anxiety
capecitabina e trastuzumabe	3	Mixed phenotype acute leukaemia, Invasive ductal carcinoma; as first-line therapy
Ciprofloxacin	4	cholangitis; Secondary prophylaxis for spontaneous bacterial peritonitis.
cisplatin	3	Pericardial effusion.
Clonazepam	1	Anxiety, dose
darunavir	3	HIV2 infection
Dienogest/etinilestradiol	1	Acne
eculizumab	1	Glomerulonephritis crescentic.
Esomeprazole plus Budesonide	1	Respiratory tract infection.
Estiripentol	2	Myoclonic epilepsy, dose and frequency increased
Goserreline	2	Uterine myoma
Ibuprofen	1	Non-productive cough.
Interferon beta-1a	1	Multiple sclerosis, paediatric use
Ketoconazole	1	Ectopic Adrenocorticotropic (ACTH) syndrome caused by a nasal paraganglioma.
mercaptopurine	1	Crohn's disease
Methotrexate	2	Eosinophilic Fasciitis, Systemic Lupus Erythematosus
Metronidazole.	2	Diarrhoea and fever in paediatric population
midostaurin	1	Population (paediatric)
Moxifloxacin plus isoniazid, pyrazinamide and ethambutol.	1	Pulmonary and Pleural Tuberculosis, resistant.
Nivolumab	2	Hepatocellular carcinoma
Paracetamol	2	Nasopharyngitis or tonsilitis.
Peginterferon beta-1a	3	Chronic Hepatitis C.
Piperacillin and tazobactam	1	Catheter-related bloodstream infection (CRBSI) caused by MRSA.
Piperacillin and Tazobactam.	1	central line infection in children
Rituximab and HAART.	1	Castleman disease and HIV infection.
Sirolimus	2	Prophylaxis against liver transplant rejection.
Sunitinib	3	Bone cancer metastatic, Metastatic thyroid cancer.
Telaprevir	1	HCV/HIV co-infection.

terbinafine	5	Onychomycosis, dose increased
ulipristal acetate 5 mg)	2	Uterus with a 12 cm volume, Multiple massive uterine fibroids without symptoms.
Ustekinumab.	4	psoriasis
Valproic acid.	1	Personality disorder.

Supplementary Table S2

Causality rates in cases of serious hepatic adverse drug reactions in relation to the number of suspected drugs

Causality	n (%)	Average number of suspected drugs	Standard Deviation
Unassigned	1303 (71.3)	2.45	2.451
Likely	273 (14.9)	1.31	0.764
Possible	202 (11.0)	1.56	0.948
Definitive	24 (1.3)	1.08	0.282
Unlikely	11 (0.6)	1.54	0.687
Conditional	7 (0.4)	1.00	0.000
Not Related	5 (0.3)	1.20	0.447
Not Classifiable	3 (0.2)	1.00	0.000

Average number and Standard deviation were used.

Supplementary Table S3

Cases with positive viral markers included in the sample of patients with reported adverse drug reactions.

Viral marker-positive	n	IH	Reactivation Relapse	Coinfection	FH	Cholestasis	Ascites	HM	FC	Other systems
CMG	6	ND	ND	1*		1		5		1
EBV	2	ND	ND				1	1		1
HBV	5	1	4		3	1		1		
HCV	10	1	5	1**			1		3	
HEV	1	ND	ND							
Herpes simplex	1	ND	ND							
Herpes zoster	3	ND	1		1		2			1
HIV	3	3	NR						1	
**Total**	**31**	**4**	**10**	**2**			**4**	**7**	**4**	**3**

Abbreviations- ND - not described; NR - not relevant; CMG – Cytomegalovirus; EBV - Epstein-Barr virus; HBV – Hepatitis B virus; HCV - Hepatitis C virus; HEV - Hepatitis E virus; HIV – human deficiency virus; n – number of cases; IH - indication for hospitalization; FH - fulminant hepatitis; HM – hepatomegaly; FC – Fibrosis Cirrhosis. HIV cases were included due to the use of antiretroviral drugs which present potential hepatotoxicity. Indication of hospitalization: report of hospitalization due to the viral infection. Reactivation relapse: report of clinical or laboratory reactivation of viral infection, including the native liver or transplanted liver. Coinfection: reported in 2 cases: *- Cytomegalovirus plus Epstein-Barr; **- HIV plus hepatitis C (patient with cirrhosis). Fulminant hepatitis: report of associated coagulation disorder or encephalopathy. Cholestasis: report of jaundice plus pruritus, choluria or acholic stools. Other systems: report of pleural or pericardial effusion, or pleuritis.

Supplementary Table S4

Table 8 - Distribution of reported cases of adverse drug reactions according to the Anatomical Therapeutic Chemical Code (ATCC)

**A Alimentary tract and metabolism n=91 (2.76%)**	**n (%)**
A01 Stomatological preparations	4 (0.12)
A02 Antacids, medicines to treat peptic ulcer and flatulence	16 (0.49)
A03 Drugs for functional gastrointestinal disorders	1 (0.03)
A05 Biliary and hepatic therapy	4 (0.12)
A06 Laxatives	1 (0.03)
A07 Antidiarrheal, intestinal anti-inflammatory and anti-infectious agents	13 (0.39)
A08 Anti-obesity preparations, excluding diet products	1 (0.03)
A10 Medicines used in diabetes	46 (1.40)
A11 Vitamins	3 (0.09)
A16 Other products for digestive tract and metabolism	2 (0.06)
**B Blood and blood forming organs n=52 (1.58%)**	
B01 Antithrombotic drugs	41 (1.25)
B02 Anti-haemorrhagic	5 (0.15)
B03 Antianemic drugs	3 (0.09)
B05 Blood substitutes and perfusion solutions	1 (0.03
B06 Other haematological products	2 (0.06)
**C Cardiovascular system n=198 (6.01%)**	
C01 Cardiac therapy	31 (0.94)
C02 Antihypertensives	31 (0.94)
C03 Diuretics	24 (0.73)
C04 Peripheral vasodilators	3 (0.09)
C05 Vasoprotectors	2 (0.06)
C07 Beta blockers	8 (0.24)
C08 Calcium channel blockers	12 (0.36)
C09 Agents that act on the renin-angiotensin system	26 (0.79)
C10 Hypolipidemic	61 (1.85)
**D Dermatologicals n=25 (0.76%)**	
D01 Antifungals for dermatological use	12 (0.36)
D05 Antipsoriatics	1 (0.03)
D06 Antibiotics and chemotherapy for dermatological use	7 (0.21)
D07 Corticosteroids, dermatological preparations	2 (0.06)
D10 Anti-acne preparations	1 (0.03)
D11 Other dermatological preparations	2 (0.06)
**G Genito urinary system and sex hormones n=50 (1.52%)**	
G01 Gynaecological anti-infectives and antiseptics	3 (0.09)
G03 Sex hormones and modulators of the genital system	36 (1.09)
G04 Urological medications	11 (0.33)
**H Systemic hormonal preparations, excluding sex hormones and insulins n=70 (2.13%)**	
H01 Pituitary, hypothalamic and analogous hormones	1 (0.03)
H02 Corticosteroids for systemic use	61 (1.85)
H03 Thyroid therapy	8 (0.24)
**J Antiinfective for systemic use n=1192 (36.20%)**	
J01 Antibacterials for systemic use	282 (8.56)
J02 Antimycotic for systemic use	41 (1.25)
J04 Antimycobacterials	131 (3.98)
J05 Antivirals for systemic use	713 (21.65)
J06 Immunosorbents and immunoglobulins	5 (0.15)
J07 Vaccines	20 (0.61)
**L Antineoplastic and immunomodulating agents n=892 (27.09%)**	
L01 Antineoplastic agents	430 (13.06)
L02 Endocrine therapy	37 (1.12)
L03 Immunostimulants	81 (2.46)
L04 Immunosuppressive agents	344 (10.45)
**M Musculo-skeletal system n=86 (2.61%)**	
M01 Anti-inflammatory and antirheumatic	50 (1.52)
M02 Topical products for joint and muscle pain	3 (0.09)
M03 Muscle relaxants	10 (0.30)
M04 Anti-gout preparations	14 (0.43)
M05 Medicines for treating bone diseases	9 (0.27)
**N Nervous system n=567 (17.22%)**	
N01 Anaesthetics	4 (0.12)

N02 Pain relievers	101 (3.07)
N03 Antiepileptics	77 (2.34)
N04 Antiparkinsonians	19 (0.58)
N05 Psycholeptics	186 (5.65)
N06 Psychoanalytic	110 (3.34)
N07 Other nervous system medications	70 (2.13)
**P Antiparasitic products, insecticides and repellents n=11 (0.33%)**	
P01 Anti-protozoa	6 (0.18)
P02 Anthelmintics	5 (0.15)
**R Respiratory system n=42 (1.28%)**	
R02 Prepared for pharyngeal use	12 (0.36)
R03 Antiasthmatics	17 (0.52)
R05 Prepared against coughs and colds	2 (0.06)
R06 Antihistamines for systemic use	11 (0.33)
**V Various n=17 (0.52%)**	
V3 Other therapeutic products	10 (0.30)
V8 Contrast media	4 (0.12)
V9 Diagnostic radiopharmaceuticals	3 (0.09)

Abbreviations: n – number of cases.
